# Chemical Analysis and Antimicrobial Activity of *Moringa oleifera* Lam. Leaves and Seeds

**DOI:** 10.3390/molecules27248920

**Published:** 2022-12-15

**Authors:** Attilio Anzano, Bruna de Falco, Mohammad Ammar, Annarita Ricciardelli, Laura Grauso, Mohammed Sabbah, Rosanna Capparelli, Virginia Lanzotti

**Affiliations:** 1 Dipartimento di Agraria, Università di Napoli Federico II, Via Università 100, 80055 Portici, Italy; 2 Canarian Science and Technology Park Foundation, Spanish Bank of Algae, University of Las Palmas de Gran Canaria, 35214 Telde, Spain; 3 Department of Nutrition and Food Technology, Faculty of Agriculture and Veterinary Medicine, An-Najah National University, Nablus P.O. Box 7, Palestine; 4 Dipartimento di Biologia, Università di Napoli Federico II, Complesso di Monte Sant’Angelo-Via Vicinale Cupa Cintia, 21, 80126 Naples, Italy

**Keywords:** *Moringa oleifera*, Moringaceae, bioactive metabolites, natural products, NMR, GC-MS, *Staphylococcus aureus*, *Staphylococcus epidermidis*, *Pseudomonas aeruginosa*, *Salmonella enterica*

## Abstract

*Moringa oleifera* is a traditional food crop widespread in Asiatic, African, and South American continents. The plant, able to grow in harsh conditions, shows a high nutritional value and medicinal potential evidencing cardioprotective, anti-inflammatory, antioxidant, and antimicrobial properties. The purpose of this study was the phytochemical analysis of *M. oleifera* and the identification of the antimicrobial compounds by combining a chemical approach with in vitro tests. The metabolite profile of *M. oleifera* polar and apolar extracts of leaves and seeds were investigated by using Nuclear Magnetic Resonance spectroscopy and Gas Chromatography-Mass Spectrometry. The antimicrobial activity of all of the obtained extract was evaluated against four bacterial pathogens (*Staphylococcus aureus*, *Staphylococcus epidermidis*, *Pseudomonas aeruginosa* and *Salmonella enterica*). The chemical analysis provided a wide set of metabolites that were identified and quantified. Moreover, apolar extracts from seeds showed a significant concentration-dependent antimicrobial activity against *S. aureus* and *S. epidermidis*, (4 mg/mL reduced the viability up to 50%) that was associated to the content of specific fatty acids. Our results remarked the advantages of an integrated approach for the identification of plant metabolites and its use in association with biological tests to recognize the compounds responsible for bioactivity without compounds purification.

## 1. Introduction

**Figure 1:**
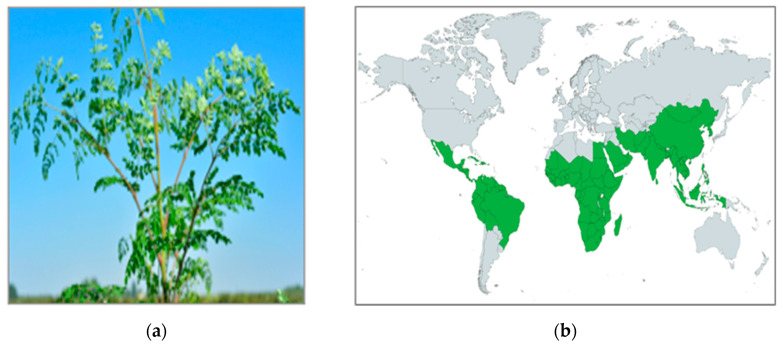
(**a**) *Moringa oleifera* tree; (**b**) Worldwide diffusion of the plant.

*Moringa oleifera* Lam., also known as miracle, horseradish or drumstick tree, is a plant member of the *Moringaceae* family able to grow as a short and slender tree. *M. oleifera* is native to Eastern countries, such as the Himalayas, India, Pakistan, Asia Minor, Africa, and Arabia [1], but today it is also distributed in other countries due to the plant tenacity making possible the cultivation in different habitats (Figure 1). The roots can penetrate deeply into the soil and retain water for long periods, making possible the growth of the plant also in dry and desertic soils and at different rainfall levels [2,3]. The optimal temperature for *M. oleifera* growth goes from 25 to 40 °C, but it can withstand temperatures ranging from −1 to 3 °C and from 38 to 48 °C, being able to resist to a large variety of environments [4].

The interest in this plant is mainly derived from its traditional uses. In fact, in traditional medicine, *M. oleifera* parts were used to treat a large variety of conditions, as the plant was believed to possess several properties, such as carminative, anti-inflammatory, laxative, anti-rheumatic activities [1,5].

Today, *M. oleifera* is used for many purposes such as in human diet and livestock feeding, thanks to the excellent nutritional aspects (high quantities of vitamins, proteins, and amino acids) [2]; in medicine, thanks to the properties mentioned earlier; as fuel wood; for soil conservation (used as green manure) and water purification (the seeds are used for clarification of water) [6].

Both polar and apolar leaf and seed extracts contain several relevant compounds belonging to the classes of fatty acids, alkanes, amino acids, glucosinolates, polyphenols, which make *M. oleifera* a very interesting plant from a nutritional and a pharmacological point of view [5,7]. Thus, several pharmacological properties have been investigated and attributed to the seed and leaf extracts, e.g., cardiovascular activity [8], anti-inflammatory activity [9], antihypertensive activity [10], radical scavenging and antioxidant activity [11,12], anticancer activity [13,14], hepatoprotective and nutraceutical activity [15,16], anti-allergic activity, antimicrobial [17] and antiviral activity [18].

The aim of this study was to explore the qualitative and quantitative aspects of the metabolite profile of *M. oleifera* leaves and seeds and to evaluate the antimicrobial activity of their polar and apolar extracts.

The interests in plant metabolite profiling approaches are rapidly growing as they can provide a wide overview of the metabolites present in a plant extract [19]. It is often used to characterize extracts from plant tissues, or to compare extracts from plants grown in different conditions, as it provides a “fingerprint” of the status of the plant.

Here, we performed an untargeted metabolite profiling analysis of leaf and seed extracts using an integrated approach of proton Nuclear Magnetic Resonance (^1^H-NMR) spectroscopy and Gas Chromatography-Mass Spectrometry (GC-MS), followed by the identification of the main components from the obtained spectra. Then, the extracts were tested against *Staphylococcus aureus*, *Staphylococcus epidermidis*, *Pseudomonas aeruginosa* and *Salmonella enterica* to assess the antimicrobial activity.

## 2. Results

### 2.1. NMR Analysis of M. oleifera Polar Extracts

Polar extracts of leaves and seeds of *M. oleifera* were analyzed in triplicate through ^1^H NMR spectroscopy obtaining a qualitative and quantitative profile of plant tissues analyzed. Peak by peak analysis of the spectra was performed, with the aid of 2D NMR experiments (COSY, HSQC and HMBC), and by comparison with standard compounds available in the laboratory and reported in the literature.

The main metabolites identified from the NMR spectra are listed in Table 1, where they are divided in chemical structural classes.

**Figure 2:**
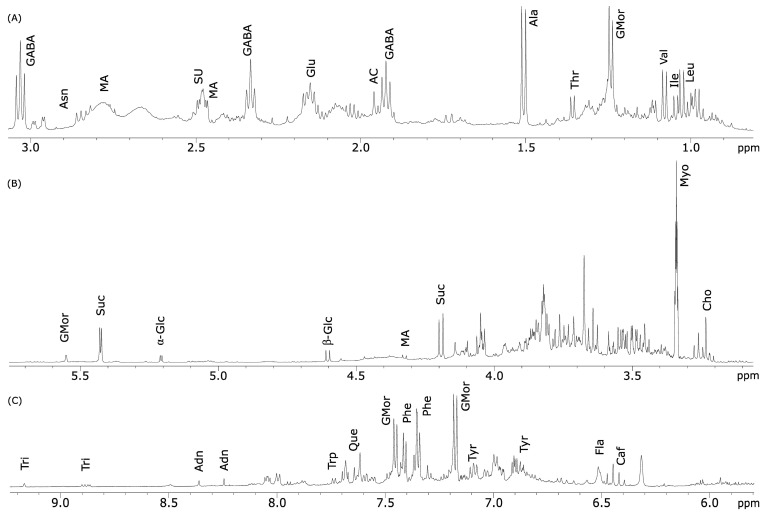
^1^H NMR spectrum of *M. oleifera* polar leaf extract acquired in D_2_O at 600 MHz. Spectral regions between (**A**) 0.5–3.1 ppm vertically expanded (×5); (**B**) 3.1–5.7 ppm; (**C**) 5.8–9.2 ppm vertically expanded (×2).

In the high field region of the leaf spectra (Figure 2A), diagnostic signals for the methyl groups of the branched amino acids are shown. Leucine (Leu), isoleucine (Ile) and valine (Val) were identified using the three doublets at δ 1.01 (J = 7.0 Hz), δ 1.05 (J = 7.0 Hz) and δ 1.08 (J = 7.0 Hz), respectively. Threonine (Thr) was also identified by a doublet at δ 1.36 (J = 7.0 Hz), corresponding to the γ-CH_3_ group, while the β-CH_3_ group of alanine (Ala) was assigned through a doublet at δ 1.51 (J = 7.0 Hz). This signal was shifted downfield compared to the previous ones because of the proximity to the nitrogen atom. Asparagine (Asn) was identified through the double doublet resonating at δ 2.98 (dd, J = 4.0 and 13.0 Hz), whereas the β-CH and β’-CH groups of glutamic acid (Glu), used for its identification, resonated as multiplets at δ 2.08 and δ 2.16, respectively. The amino acid γ-aminobutyrate (GABA) was identified using the triplet at δ 3.04 (J = 7.0 Hz).

In the low field region of the ^1^H NMR spectra of leaves (Figure 2C), all of the characteristic groups belonging to aromatic amino acids were found. Going into detail, the multiplet at δ 7.45 and the triplet at at 7.35 were assigned to phenylalanine (Phe), the doublets at δ 6.86 and 7.10 belonged to tyrosine (Tyr) and the doublet at δ 7.74 (J = 7.5 Hz) was indicative of tryptophan (Trp), all of them corresponding to aromatic protons at different positions of the skeleton (see Table 1).

The signals used for the identification of five organic acids were found across all of the spectral regions (Figure 2A–C). Acetic acid (AC) was identified through the singlet at δ 1.96 found in the high field region of the spectra and corresponded to the α-CH_3_ group. Malic acid (MA) was identified by a double doublet resonating at δ 4.33 (J = 8.9 and 3.7 Hz), while fumaric acid (FU) showed the characteristic singlet at δ 6.61, both of them being associated to a α-CH group. Signals detected and associated to citric acid (CI) and succinic acid (SU), resonating, respectively, at δ 2.50 and 2.60, were overlapped, and the quantification of these two metabolites was not possible.

The region of the spectra between δ 3.35 and δ 4.10 (Figure 2B) was very crowded due to the high number of signals belonging to sugars and sugar alcohols that were not useful for compound identification. The characteristic signal of the anomeric proton (H-1) of α-glucose (α-Glc) resonated at δ 5.21 (d, J = 4.0 Hz), while the anomeric proton of β-glucose (β-Glc) appeared at δ 4.60 (d, J = 8.0 Hz) (Figure 2B and Table 1). Sucrose (Suc) was identified using the anomeric proton signal resonating at δ 5.43 (d, J = 3.8 Hz) due to its glucose moiety. The triplet resonating at δ 3.34 (J = 9.5 Hz) was assigned to myo-inositol (Myo).

Among other compounds, choline (Cho) presence was showed by the characteristic singlet corresponding to its methyl groups, resonating at δ 3.24, and shifted downfield in the spectra because of the presence of nitrogen (Table 1). Glucomoringin (GMor) was identified using the doublet (J = 2.0 Hz) resonating at δ 5.57, corresponding to H-1 of its rhamnose residue.

In the low-field region between 5.7 and 9.2 ppm, caffeic acid (Caf) was identified by a doublet resonating at δ 6.42 (J = 16.0). Signals for flavonoids (Fla) and in particular for quercetin (Que) were found at δ 6.53 and δ 7.65, respectively; both of them were only present in spectra of leaves. Lastly, the nucleoside adenosine (Adn) showed a singlet at δ 8.36 (s, CH-8), while the presence of trigonelline (Tri) was revealed by the characteristic signals resonating at δ 9.17 (s, CH-1) and 8.88 (t, CH-3,5) (Figure 2C and Table 1).

**Figure 3:**
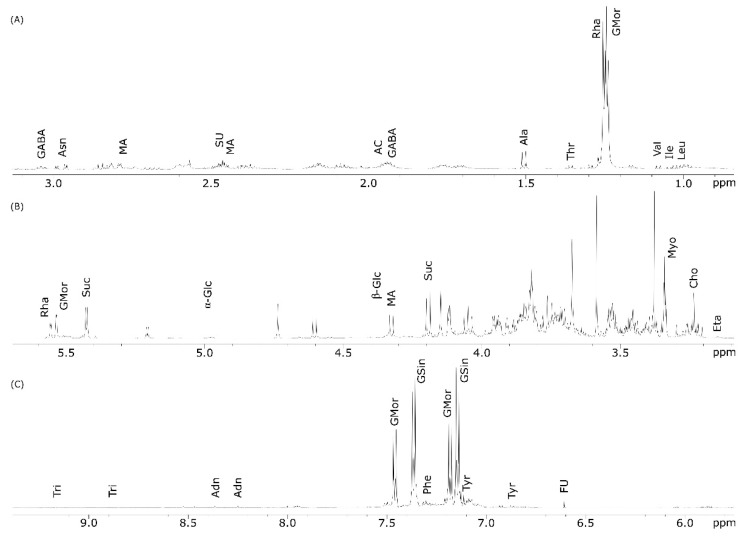
^1^H NMR spectrum of *M. oleifera* polar seed extract run in D_2_O at 600 MHz. Spectral regions between (**A**) 0.5–3.1 ppm; (**B**) 3.1–5.7 ppm; (**C**) 5.8–9.3 ppm vertically expanded (×3).

Figure 3 shows the ^1^H NMR spectrum of the seeds polar extract with compound identification. A close similarity with the NMR spectrum of leaves (Figure 2) was evident, although there were some differences in the metabolite profiles (Table 1).

Going into detail, signals for flavonoids and, particularly, quercetin were undetectable as well as that for caffeic acid (Figure 3). The amino acids phenylalanine and glutamic acid were not found in the seed extracts, while the signal identifying fumaric acid were additionally present.

The aromatic amino acids were undetectable in the seed extracts. Interestingly, in the aromatic region, additional signals at δ 7.15 and 7.37 (each d, J = 7.0 Hz) appeared; this could be attributed to glucosinolates (GSin) whose presence was confirmed by the signals at δ 5.55 and 5.53 corresponding to H-1 of rhamnose (Figure 3B). Among other compounds, ethanolamine (Eta) was identified using the broad triplet resonating at δ 3.15 (β-CH_2_, J = 7.0) (Table 1).

**Figure 4:**
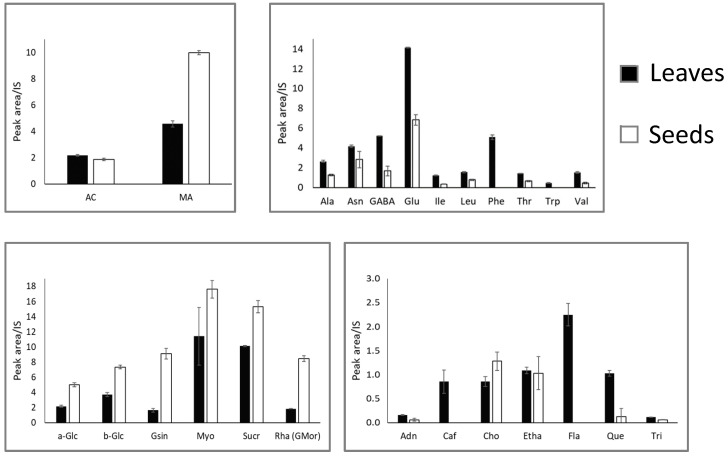
Quantitative data of the metabolites identified in leaves and seeds polar extracts. Top-left, organic acids; top-right, amino acids; bottom-left, sugars; bottom-right, other compounds. Shown data refers to the mean and standard deviation of three replicates.

The differences in the quantified metabolites between leaf and seed extracts are shown in Figure 4. Acetic acid and malic acid were the only two organic acids that could be quantified. Acetic acid was slightly more abundant in the leaf extracts. Malic acid quantity was roughly double in seeds compared to the malic acid content of leaves.

All of the amino acids were more abundant in leaf extracts than in seed extracts. Glutamic acid was the amino acid present in the larger quantity, and along with phenylalanine they showed the largest difference between the two extracts. The aromatic amino acids phenylalanine and tryptophane were undetectable in seed extracts. (Figure 4).

Conversely, all of the carbohydrates were more abundant in seeds, especially for glucosinolates and glucomoringin, that showed the largest difference among the two extracts. Monosaccharides and disaccharides followed the same trend, but the difference was smaller. Considering the relative and the absolute quantity, carbohydrates were also the most abundant molecules in the leave and seed extracts overall.

Regarding the other compounds, caffeic acid and flavonoids were only detected in leaf extracts. Moreover, the quantity of quercetin was larger in leaf extracts, while ethanolamine and trigonelline showed a comparable quantity in the two extracts. Finally, choline was present in a larger amount in the seed extracts.

### 2.2. GC-MS Analysis of M. oleifera Apolar Extracts

**Figure 5:**
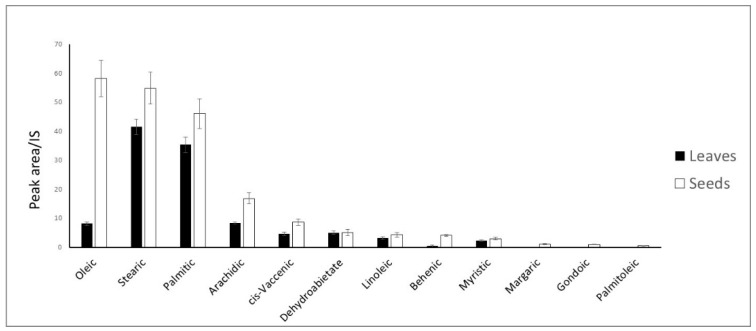
Quantitative data of the metabolites identified in leaves and seeds apolar extracts. Shown data refers to the mean and standard deviation of three replicates.

GC-MS spectrometry was used to analyze the apolar extracts from leaves and seeds from a qualitative and quantitative point of view. All of the identified metabolites were present in a larger quantity in apolar seed extract compared to apolar leaf extract (Figure 5).

Oleic acid is the one present in the largest amount in the apolar seed extract, followed by stearic acid and palmitic acid. Oleic acid is also the fatty acid that shows the largest concentration difference between leaf and seed apolar extracts. On the contrary, stearic acid was the most abundant fatty acid in leaves, followed by palmitic acid. Arachidic acid was also quite abundant, especially for seed apolar extract.

All of the other detected fatty acids, including cis-vaccenic, linoleic, behenic, myristic, lauric, margaric, gondoic, palmitoleic, and lignoceric acid, were present in minor amounts. Gondoic and palmitoleic acid were only detected in seed extracts.

Oleic acid represents the major metabolite among the unsaturated fatty acids in the seeds (58.18 ± 6.32), while it accounted only for 8.17 ± 0.57 in the leaves. (Figure 5). In addition to fatty acids, two terpenoids were identified as methyl abiet-8-en-18-oate, present only in leaves, and methyl dehydroabietate, detected in both apolar extracts.

### 2.3. Antimicrobial Activity

**Figure 6:**
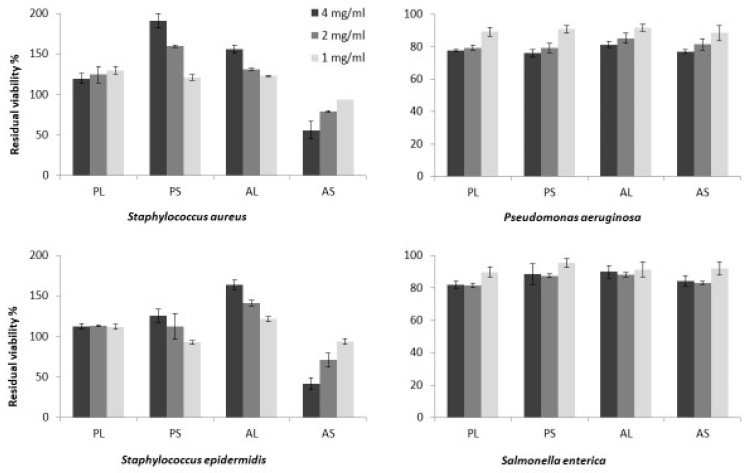
Antimicrobial activity of the *M. oleifera* extracts: PL = polar leaves extract; PS = polar seeds ectract; AL = apolar leaves extract; AS = apolar seeds extract, tested at 4 mg/mL, 2 mg/mL and 1 mg/mL. Bacterial viability was assessed by measuring the optical density at 600 nm and expressed as percentage of residual viability compared to the untreated samples. Showed data refers to the mean and standard deviation of three replicates.

Polar and apolar extracts obtained from the leaves and seeds of *M. oleifera* were tested for antimicrobial activity against two Gram-positive (*Staphylococcus aureus* and *Staphylococcus epidermidis*) and two Gram-negative (*Pseudomonas aeruginosa* and *Salmonella enterica*) pathogens (Figure 6).

In detail, microbial cultures of all of the tested pathogens were incubated for 24 h in presence and in absence of extracts at 37 °C. Then, the residual viability of treated cultures was evaluated in comparison to the respective untreated samples. Data reported in Figure 6 show that neither polar nor apolar extracts obtained from leaves and seeds significantly affected the microbial growth of the tested Gram-negative bacteria, *P. aeruginosa* and *S. enterica*. Whereas, among all, only apolar extracts obtained from seeds (AS) showed a clear antimicrobial activity against the tested Gram-positive pathogens (Figure 6). Moreover, AS induced a reduction in microbial viability in a dose-dependent manner, with an effectiveness of around the 50% at a concentration of 4 mg/mL, on both the tested *Staphylococcus* species.

## 3. Discussion

Chemical characterization of *M. oleifera* was carried out using an integrated approach based on ^1^H NMR and GC-MS analyses.

Regarding the polar fraction extracted from leaves and seeds, twenty-nine metabolites were identified and quantified (Table 1 and Figure 4). The main metabolites are amino acids, eleven of which were annotated. Generally, they were more abundant in leaves than in seeds. Six carbohydrates were identified and all of them were more abundant in seed than in leaf extracts. Only five organic acids were identified, but signals for succinic and acetic acids were overlapped and, thus, only three of them were quantified. Lastly, among other compounds adenosine, caffeic acid, choline, ethanolamine, quercetin, trigonelline and the general class of the flavonoids were identified and quantified, and all of them were more abundant in leaves except for choline. The chemical composition found by the methods used in this study is similar to the one found by a previous article [20].

The GC-MS analysis resulted in the identification of twelve metabolites, all of them being more present in seeds than in leaves (Figure 5). Oleic acid is the main fatty acid, six times more abundant in seeds than in leaves, and with cis-vaccenic, linoleic, gondoic and palmitoleic acid, it represents the unsaturated fraction of the oil. Conversely, the saturated fraction is composed of stearic, palmitic, arachidic, behenic, myristic and margaric acid.

The antimicrobial assay showed the apolar fraction of the seeds exerts significant antimicrobial activity against the Gram-positive bacteria *S. aureus* and *S. epidermidis*, while it was not active against *S. enterica*. and *P. aeruginosa* (Figure 6). These results agree with the literature [21,22] reporting on antimicrobial activity for *M. oleifera* seed oil against *S. aureus* [21]. Similar activity has been reported for *Moringa peregrina* oil [22]. Conversely, no antimicrobial effects were found for aqueous extracts from leaves [23]. However, studies have detected antimicrobial activity for polar extracts from leaves and seeds, but at concentrations higher than the ones used in our study [24,25,26]. Moreover, other studies found no antimicrobial effects against *S. aureus* for seeds petroleum ether extract [27,28].

The different effects between the apolar fractions of seeds and leaves could be due to the remarkable difference in fatty acids content of the two fractions. It is well known that free fatty acids and monoglycerides can exert an antimicrobial effect, especially against Gram-positive bacteria [29,30,31,32] that is dependent on the number of double bonds, since unsaturated fatty acids seem to be more active than saturated, and there is a correlation between the number of double bonds possessed by a fatty acid and its antimicrobial activity [31]. The antimicrobial activity of fatty acids also depends on the length of the carbon chain. The mechanism of action is still under debate, but it could be due to the detergent properties of free fatty acids that can be disruptive for the cellular membrane integrity or for the functionality of the enzymes involved in the electron transport chain.

## 4. Materials and Methods

### 4.1. Chemicals and Solvents

n-hexane and methanol were obtained from Delchimica Scientific Laboratories (Naples, Italy). Deuterium oxide (D_2_O, 99.8 atom% D), used in NMR experiments, was obtained from ARMAR Chemicals (Döttingen, Switzerland). Dimethyl—4—silapentane sodium sulphonate (DSS), used in NMR experiments, was purchased from Merck (Darmstadt, Germany).

### 4.2. Plant Material

*Moringa oleifera* dried leaves and seeds were bought from a local shop, in Nablus, Palestine. Leaves and seeds were grinded using a kitchen mixer to obtain a fine powder that was subjected to further extraction procedure and analyses.

### 4.3. Metabolite Extraction

The metabolite extraction was performed following the method described in de Falco et al. [33]. A total of 4 g of powdered leaves and seeds were extracted with 50 mL of n-hexane, stirring the solution for 1 h. Then, the apolar extracts obtained were filtered, transferred into vials, and dried at room temperature. The remaining pellets were subjected to polar extractions using 50 mL of a methanol/water solution (1:1), stirring the solution for 1 h. The supernatants were then separated by filtration and centrifuged at 3000 rpm for 10 min, RT. The extracts were then dried using a rotary evaporator (30 °C). Both apolar and polar extracts were stored at 4 °C waiting for further analyses. The procedure was performed in triplicate for each plant material analyzed.

### 4.4. ^1^H-NMR Analysis

A total of 10 mg of the polar extracts were solubilized in 600 μL of D_2_O and transferred into a 5 mm NMR tube. Then DSS was added as internal standard at a concentration of 0.2 mg/mL. The NMR spectra were recorded at 298 K on a Varian Unity Inova spectrometer operating at 600 MHz. The ^1^H-NMR experiments were performed with 128 transients and 16 K complex data point. The recycle time was set to 5 s, and a 45° pulse angle was used. Chemical shifts were referred to DSS signal (Δ 0.00 ppm). All spectra were processed using iNMR program (www.inmr.net, Zimmar Holdings Ltd Company, Westmount, Canada), phased and baseline corrected. In total, 12 spectra were acquired. Quantification was performed by signal integration relative to the internal standard, DSS, as described in Lanzotti et al. [34]. The region of the solvent peaks was excluded from the analysis. Spectral peak assignments of the detected compounds were obtained based on pure standards purchased by Sigma-Aldrich, Saint Louis, MO, USA, and on combined comparison with data reported in the literature and in Human Metabolome Database (HMDB). All spectra were manually phased and baseline corrected.

### 4.5. GC-MS Analysis

To obtain stable and volatile compounds, apolar extracts were derivatized by using a methanolysis reaction before GC-MS analysis. For this purpose, an aliquot of each apolar extract (0.5 mg) was transferred into a vial and dissolved in 1 mL of methanol + hydrochloric acid 1 N. The vials were vortexed and left at 50 °C overnight, then the reaction mixtures were dried under nitrogen, solubilized in n-hexane and analyzed by GC-MS. According to the method described in Grauso et al. [35]. 1 μL of derivatized samples were injected in a pulsed splitless mode into an Agilent—7820A GC system with 5977E MSD operating in electrospray ionization (EI) mode at 70 eV [35]. The system was equipped with a 30 m × 0.25 mm inner diameter (i.d.) fused—silica capillary column with 0.25 μm HP—5MS stationary phase (Agilent Technologies, Cheadle, UK). The injection temperature was set at 270 °C. Helium was used as carrier gas at a constant flow rate of 1 mL/min. Separation of the apolar extract was achieved using a temperature program of 80 °C for 1 min, then ramped at 10 °C/min to 320 °C and held for 1 min. Both chromatograms and mass spectra were evaluated using the MassHunter Qualitative Analysis B.07.00 (Agilent Technologies, Santa Clara, CA, USA). Mass spectra of all detected compounds were compared with fatty acid methyl esters (FAME) as standard compounds and with spectra obtained by the National Institute of Standard and Technologies library NIST MS search. Data were processed with the AMDIS (Agilent Technologies) software to deconvolute co-eluting peaks. The relative amounts of separated metabolites were calculated from total ion chromatography (TIC) by the computerized integrator and by comparison with internal standard, 1—oleoyl—rac—glycerol, added to the apolar extract as described in Grauso et al. [36].

### 4.6. Antimicrobial Activity Assay

The study included the following species: *Stapyloccocus aureus*, *Staphylococcus epidermidis*, *Pseudomonas aeruginosa* and *Salmonella enterica*. Isolates were obtained from patients hospitalized at the Medical School of the University of Naples Federico II. Specimens were analyzed using PCR assay as described in Romanelli et al. [37].

The antimicrobial effect of *M. oleifera* polar and apolar extracts from leaves (PL, AL) and polar and apolar extracts from seeds (PS, AS) were evaluated. Briefly, the wells of a sterile 96-well flat-bottomed polystyrene plate were filled with 200 μL of bacterial culture diluted in Tryptic Soy Broth (TSB) to a final concentration of 1 × 10^6^ colony forming units (CFU) mL^−1^ in absence and in presence of tested extracts. Each extract was first solubilized in dimethyl sulfoxide (DMSO) and then added to the culture medium and tested at concentrations of 4 mg/mL, 2 mg/mL and 1 mg/mL (DMSO final concentrations ≤ 2.5% *v*/*v*). Proper negative controls with only DMSO were included in the experiments. After 24 h incubation at 37 °C, the antimicrobial activity was optically evaluated comparing treated and untreated samples by measuring microbial growth at 600 nm wavelength with a microplate reader.

## 5. Conclusions

The chemical analysis on polar and apolar extracts of *M. oleifera* leaves and seeds allowed for the characterization of the metabolite profile, and the further identification and quantification of several primary and secondary metabolites by using a combination of ^1^H NMR and GC-MS analyses. Moreover, both polar and apolar extracts from leaves and seeds were tested to assess their antimicrobial activity. Only the seeds apolar extracts showed a dose-dependent inhibition against *S. aureus* and *S. epidermidis*, and it seems to be due to the remarkably different fatty acid content the two apolar extracts. These findings will be useful for further studies aimed to explore the many other biological potential of this very interesting plant.

## Figures and Tables

**Table 1:** ^1^H NMR chemical shifts, assignment, and multiplicity at 600 MHz in D_2_O of organic compounds detected in the polar extracts of *M. oleifera* leaves and seeds.

Compound	Assignment	^1^H (ppm)	Multiplicity (J in Hz)	Leaves	Seeds
Organic acids					
Citric acid (CI)	α,γ-CH_2_	2.50 **	d (15.0, 15.0)	x	x
	α’,γ’-CH_2_	2.68 **	d		
Fumaric acid (FU)	α-CH	6.61 *	s		x
Malic acid (MA)	β’-CH_2_	2.43	dd (15.7, 8.9)	x	x
	β-CH	2.78	dd (15.7, 3.7)		
	α-CH	4.33 *	dd (8.9, 3.7)		
Succinic acid (SU)	α-CH_2_	2.42 **	s	x	x
Acetic acid (AC)	α-CH_3_	1.96 *	s	x	x
Amino acids					
Alanine (Ala)	β-CH_3_	1.51 *	d (7.0)	x	x
Asparagine (Asn)	β-CH	2.84	dd (17.4, 3.8)	x	x
		2.98 *	dd (4.0, 13.0)		
Isoleucine (Ile)	δ-CH_3_	0.93	t (7.0)	x	x
	γ’-CH_3_	1.05 *	d (7.0)		
γ-aminobutyrate (GABA)	β-CH_2_	1.92	m	x	x
	α-CH_2_	2.34	t (7.0)		
	γ-CH_2_	3.04 *	t (7.0)		
Leucine (Leu)	δ-CH_3_	1.01 *	d (7.0)	x	x
Glutamic acid (Glu)	β, β’-CH	2.08, 2.16 *	m	x	
Phenylalanine (Phe)	CH-4	7.35	t (7.0)	x	x
	CH-2,6	7.45 *	m		
Threonine (Thr)	γ-CH_3_	1.36 *	d (7.0)	x	x
Tryptophane (Trp)	CH	7.74 *	d (7.5)	x	
Tyrosine (Tyr)	CH-3,5	6.86 **	d (7.0)	x	x
	CH-6,8	7.10 **	d (7.0)		
Valine (Val)	γ’-CH_3_	1.03	d (7.0)	x	x
	γ-CH_3_	1.08 *	d (7.0)		
Carbohydrates					
β-Glucose (β-Glc)	CH-1	4.60 *	d (8.0)	x	x
α-Glucose (α-Glc)	CH-1	5.21 *	d (4.0)	x	x
Sucrose (Suc)	Glc CH-1	5.43 *	d (3.8)	x	x
	Fru CH-3′	4.20	d (8.5)		
myo-Inositol (Myo)	CH-4	3.34 *	t (9.5)	x	x
	CH-2,5	3.58 **			
	CH-3,6	3.66 **			
Other compounds					
Adenosine (Adn)	CH-2	8.25	s	x	x
	CH-8	8.36 *	s		
Caffeic acid (Caf)	α-CH	6.42 *	d (16.0)	x	
Flavonoids (Fla)	CH	6.53 *		x	
Choline (Cho)	N(CH_3_)_3_^+^	3.24 *	s	x	x
Ethanolamine (Eta)	β-CH_2_	3.15 *	bt (7.0)		x
Glucomoringin (GMor)	CH_3_	1.19	d	x	x
	Glc CH-1	4.33	d (8.0)		
	Rha CH-1	5.57 *	d (2.0)		
Glucosinolates (GSin)	Rha CH-1	5.55 *	d (2.0)		x
	Rha CH-1	5.53 *	d (2.0)		
Quercetin (Que)	CH	7.65 *		x	
Trigonelline (Tri)	CH-4	8.12	t	x	x
	CH-3,5	8.88	t		
	CH-1	9.17 *	s		

* Signal used for quantification. ** Signal partially overlapped.

## Data Availability

Not applicable.

## Sample Availability

Samples of *M. oleifera* extracts are available from the authors.
